# Open-label, phase I dose-escalation study of sodium selenate, a novel activator of PP2A, in patients with castration-resistant prostate cancer

**DOI:** 10.1038/sj.bjc.6605798

**Published:** 2010-07-20

**Authors:** N M Corcoran, C M Hovens, M Michael, M A Rosenthal, A J Costello

**Affiliations:** 1 Department of Surgery, Division of Urology University of Melbourne, Royal Melbourne Hospital, 5th Floor Clinical Sciences Building, Royal Parade, Parkville, Victoria 3050, Australia; 2 Division of Haematology and Medical Oncology, Peter MacCallum Cancer Centre, Locked Bag 1, A’Beckett Street, Melbourne, Victoria 3002, Australia; 3 Department of Medical Oncology, Royal Melbourne Hospital, Grattan Street, Parkville, Victoria 3050, Australia

**Keywords:** prostate cancer, sodium selenate, PP2A phosphatase, anti-angiogenesis, phase I study

## Abstract

**Background:**

Angiogenesis is fundamental to the progression of many solid tumours including prostate cancer. Sodium selenate is a small, water-soluble, orally bioavailable activator of PP2A phosphatase with anti-angiogenic properties.

**Methods:**

This was a dose-escalation phase I study in men with asymptomatic, chemotherapy-naïve, castration-resistant prostate cancer. The primary objective was to determine the maximum tolerated dose (MTD). Secondary objectives included establishing the safety, tolerability and pharmacokinetic profile.

**Results:**

A total of 19 patients were enrolled. The MTD was 60 mg per day. Dose-limiting toxicity (fatigue and diarrhoea) was observed at 90 mg per day. The most frequently reported treatment-related adverse events across all treatment cohorts were nausea, diarrhoea, fatigue, muscle spasms, alopecia and nail disorders. No grade 4 toxicities were observed and there were no deaths on study. Linear pharmacokinetics was observed. One patient had a PSA response >50%. Median time to PSA progression (for non-responders) was 14.2 weeks. Mean PSA doubling time increased during the main treatment phase from 2.18 months before trial to 3.85 months.

**Conclusion:**

Sodium selenate is well tolerated at a dose of 60 mg per day with modest single-agent efficacy similar to other anti-angiogenic agents. Further trials in combination with conventional cytotoxic regimens are warranted.

Prostate carcinoma is a common cancer, which is lethal in up to 15% of patients (Baade *et al*, 2004; Jemal *et al*, 2009). The vast majority of patients die from castrate-resistant disease, evidenced by continued tumour progression despite low levels of circulating androgens. The ability of traditional cytotoxic agents to affect meaningfully on survival is limited, with the most efficacious docetaxel-based regimens extending median survival by just more than 2 months. There is thus an urgent need for new agents that either significantly affect the disease as a stand-alone agent or sensitise tumours to existing therapies.

The ability to independently initiate new blood vessel formation is a ‘hallmark’ of solid tumours. The importance of angiogenesis in the development and progression of prostate cancer is underscored by numerous studies evaluating microvessel density (MVD), a histological correlate of angiogenic potential, in human prostate samples. The MVD of prostate cancer is consistently higher than normal tissue, and is positively associated with more advanced tumour stage, higher Gleason scores, and the presence of metastatic disease. In addition, MVD predicts biochemical recurrence after both radical prostatectomy and external beam radiotherapy (Halvorsen *et al*, 2000; Revelos *et al*, 2007), and when used with other markers of angiogenic potential, predicts overall survival (Mucci *et al*, 2009). Similarly, significantly higher levels of VEGF, the predominant pro-angiogenic signalling molecule, are detected in patients with metastatic disease, and in hormone refractory prostate cancer, when both serum and urine levels predict survival (Bok *et al*, 2001; George *et al*, 2001). Over the last two decades the role of protein kinases in co-ordinating cancer-related neo-angiogenesis through the ‘switch-like’ reversible phosphorylation of key regulatory molecules in response to pro-angiogenic signals has been extensively elaborated (Ahmed and Bicknell, 2009; Aragon-Ching and Dahut, 2009). On the basis of this knowledge a number of antibodies and small-molecule kinase inhibitors that target pro-angiogenic signalling have been developed, and have been successfully translated to clinical use in renal, breast, non-small cell lung, colorectal and hepatic carcinoma (Ivy *et al*, 2009). At the same time, relatively little attention has been paid to the role of protein phosphatases, molecules that promote subsequent dephosphorylation, in limiting or even suppressing pro-angiogenic signalling. We have identified a specific selenium-containing compound, sodium selenate, which significantly boosts the activity of a key intracellular phosphatase, PP2A (Corcoran *et al*, 2010). PP2A has been shown to negatively regulate a number of intracellular proteins that are important in angiogenic signalling, particularly components of the PI3K/Akt and MAPK pathways, in endothelial cells (Young *et al*, 2002; Young, 2004; Kim *et al*, 2009). We have previously shown that sodium selenate inhibits experimental tumour growth in an orthotopic model of castration-resistant prostate cancer (CRPC) by inhibiting vessel branching (Corcoran *et al*, 2004). This first-time-in-human study examined the novel PP2A stimulator, sodium selenate, in men with CRPC.

The primary aims of this study were to assess the safety, tolerability and pharmacokinetics of sodium selenate in men with CRPC.

## Patients and methods

### Patient selection

Patients with CRPC defined by at least three successive rises in serum PSA at least 2 weeks apart, in the presence of castrate levels of serum testosterone, were eligible for enrolment in this study. Anti-androgen therapy must have been discontinued at least 4 weeks before entry into the trial, with evidence of continuing PSA rises after this time, with a PSA level ⩾5 *μ*g l^–1^ required at study entry. LHRH agonists were continued and allowed concurrently. In addition, patients were required to be asymptomatic or have only minor symptoms due to their prostate cancer, have a WHO performance status ⩽2, and an estimated life expectancy of at least 6 months. Patients were also required to have adequate renal (serum creatinine <1.5 ULRR), liver (serum bilirubin <1.25 and AST/ALT <2.5 ULRR) and haematological reserve (absolute neutrophil count >1 × 10^9^ l^–1^ and platelets >100 × 10^9^ l^–1^), and have no evidence of severe or uncontrolled systemic disease.

Patients treated previously with cytotoxic chemotherapy or strontium therapy, or who had coexisting malignancies or malignancies diagnosed within the last 5 years (with the exception of non-melanomatous skin cancer), were ineligible for enrolment. Treatment with any investigational agent within 4 weeks of study entry was not allowed, and patients with unresolved chronic toxicity greater than Common Terminology Criteria grade 2 from previous anti-cancer therapy or incomplete healing from previous surgery were excluded. Although the previous use of commercially available selenium supplementation was not a specific exclusion criterion, concomitant use over the study period was not permitted. Similarly, the concomitant use of unapproved or herbal remedies for prostate cancer was prohibited during the study period.

The study received institutional review board approval and all patients gave written informed consent.

### Study design and treatment plan

This was an open-label phase I dose-escalation study. After signing informed consent, patients underwent baseline testing to confirm eligibility. Patients who satisfied the inclusion and exclusion requirements were invited to enter the study according to the dose-escalation criteria. Baseline evaluations were conducted within 2 weeks of starting therapy. Sodium selenate was administered daily for 3 weeks (one cycle). After four cycles of therapy (12 weeks), patients with stable or responding disease, and who wished to continue on study, were offered ongoing treatment for a further 12 weeks. During the main treatment phase (weeks 0–12) patients underwent assessments for safety and tolerability at the end of each cycle. Patients opting to continue the drug beyond this time were similarly evaluated at six weekly intervals. All patients were assessed for safety 28 days after the last dose of study drug, and where possible, all patients were evaluated 3 months after their final treatment with sodium selenate.

At study commencement, sodium selenate was administered as an oral capsule taken once a day on an outpatient basis. Patients were given a fixed dose and there was no intra-patient dose-escalation. After the initial pharmacokinetic analysis demonstrated a short drug half-life in serum, the same total daily dose was given in three divided doses in one 24-h period to generate steady-state plasma selenate levels (beginning with patient 5).

For the initial dose levels, an accelerated titration design was used with one patient at each dose (5 mg, 10 mg, 15 mg, 30 mg) treated and evaluated at the end of 3 weeks. Toxicities were graded according to Common Terminology Criteria for Adverse Events version 3. Drug-related toxicity (DRT) was defined as any grade 2 non-haematological toxicity and/or any grade 3 haematological toxicity. In the absence of a DRT, commencement of a patient at the next dose level occurred. Three patients were subsequently enrolled to each fixed-dose cohort, with planned dose levels of 60, 90 and 120 mg per day. The protocol was later amended to include 45 mg per day to collect additional safety information. Dose-escalation continued until dose-limiting toxicity (DLT) was observed in one-third of patients during cycle 1. The DLT was defined as any grade 3/4 non-haematological toxicity and/or and grade 4 haematological toxicity. If DLT was observed in one of three patients in a cohort, a further three patients were recruited to that dose level. If one further patient experienced DLT, then this defined the DLT level. If no DLT was observed, patients were recruited to the next dose level. The maximum tolerated dose (MTD) was defined as the dose level below that in which ⩾33% (2 of 6) of patients were observed to have a DLT. The MTD was then expanded to six patients.

If patients developed ⩾ grade 2 haematological and/or non-haematological toxicity during the main treatment phase, sodium selenate was not administered. If treatment was deferred because of toxicity, it could be re-initiated once toxicity was ⩽ grade 1. Treatment delays of up to 2 weeks were allowed, but no dose reductions were permitted. In the absence of treatment delays due to adverse events, treatment continued for up to four cycles or until the disease progressed, the occurrence of an inter-current illness that prevented further administration of treatment, or the patient decided to withdraw from the study. In the absence of toxicity, patients were allowed to remain on selenate beyond the main treatment phase if they chose to do so.

### Drug product

Sodium selenate was blended with the inert pharmaceutical excipient lactose and filled into capsules using a Dott Bonapace (Milan, Italy) semi-automatic capsule filler, under GMP conditions, using the appropriate size capsules. Three dosage strengths were prepared, containing 5, 20 or 50 mg of sodium selenate. Independent quality control testing was performed on the finished product, and stability at 25°C for the time course of the study confirmed.

### Patient evaluation

Before registration, a complete medical history and physical examination were performed, castrate levels of serum testosterone confirmed, and baseline haematology and biochemistry were obtained. Patients were then evaluated at the start of each treatment cycle for possible adverse events or toxicity (performance status, physical examination, electrocardiogram, full blood count, urea and electrolytes, liver function tests). All patients entering the trial had an additional safety visit at 4 and 12 weeks after completion of therapy to assess for residual toxicity. To assess for tumour response, serum PSA was measured at the completion of each treatment cycle. Although the trial was initiated before the publication of the Prostate Cancer Trials Working Group 2 criteria for PSA end points, PSA data are analysed according to their recommendations (Scher *et al*, 2008). In particular, the percentage change in PSA from baseline to the completion of initial 12-week evaluation, or date of withdrawal from study if earlier, is presented in a waterfall plot. For patients experiencing an initial decline in PSA, time to PSA progression is defined by the date of documentation of PSA ⩾25% above nadir with an absolute increase of ⩾2 ng ml^–1^ confirmed on a second reading ⩾3 weeks later. For patients not experiencing a decline in baseline PSA, the date of progression is defined by the date of documentation of PSA ⩾25% with an absolute increase ⩾2 ng ml^–1^ above baseline, after 12 weeks on treatment. In addition, PSA doubling time was calculated using the log-slope method as previously described (Daskivich *et al*, 2006). For pre-trial PSA measurements, a weighted linear regression was performed, with PSA measurements >16 weeks before study entry discounted by 50%. All other PSA values, including those on trial, were given equal weighting (weight=1). To calculate the mean cohort doubling time, the slopes of individual regression equations were averaged and its inverse multiplied by the log of 2.

### Pharmacokinetic studies

Blood samples were obtained from all patients during their first course of treatment, at the following time points: pre-dose (within 60 min of drug administration), post-dose at 30 min, 1, 2, 4, 6, 24, 48 and 72 h. In addition, trough samples were taken at days 7, 14 and 21. Samples of 10 ml were collected into potassium–EDTA tubes from an indwelling arm vein catheter, and centrifuged at 2000 **g** at 4°C for 15 min. The plasma was immediately stored at −80°C. Urine was collected over a 24-h period immediately after the first dose on day 1. The 24-h urine sample was thoroughly mixed, volume recorded and a 6-ml aliquot was taken and stored at −80°C. Samples were analysed for sodium selenate (parent) and its various metabolites using ion chromatography dynamic reaction cell inductively coupled plasma mass spectrometry (Kuo and Jiang, 2008).

### Pharmacokinetic analysis

Pharmacokinetic results were processed according to standard non-compartmental analytical procedures (Win Non Lin v5.2, Pharsight Corporation, Mountain View, CA, USA). The actual times of sample collections were used in the calculations. Parameters measured included *C*_max_, *C*_min_, *T*_max_ and AUC_last_. AUC was determined by the linear trapezoidal rule, where AUC_last_ was the AUC from time zero until the last concentration point. The elimination rate constant, Kel (h^−1^) was determined by linear regression of a minimum of three points. Half-life (*t*_1/2_) was determined according to *t*_1/2_=0.693 Kel^–1^.

### Statistical evaluations

All data presented are descriptive and no formal statistical evaluation was performed.

## Results

### Patient characteristics

Between June 2006 and November 2008, a total of 19 patients were enrolled in the study and their clinical characteristics are summarised in Table 1. The median age was 72 years and the mean PSA was 22 *μ*g l^–1^, with an average PSA doubling time of 2.2 months (1.1–8.9). In all, 7 of 19 patients (36.9%) had previously received treatment to the primary tumour, three with radical surgery and four with external beam radiotherapy. The remaining 12 patients had androgen deprivation as their only prostate cancer treatment. In all, 10 of 19 patients (52.6%) had metastatic bone disease at the time of enrolment.

### Safety results

Of the 19 patients enrolled into the study, 12 patients (63%) completed the 12-week treatment period (Table 2). In all, 6 of the 12 patients who completed the 12-week study period continued to receive study drug beyond this time for a total treatment period ranging from 23–28 weeks. Seven patients withdrew from the study drug before completion of the main treatment phase due to disease progression (four patients), unacceptable toxicity (two patients, one with grade 3 fatigue and one with concomitant grade 3 diarrhoea and muscle cramps) or the occurrence of an adverse event (one patient with acute renal impairment)

Table 3 documents the toxicities observed during the study thought to be at least possibly related to sodium selenate. The majority (76%) were classified as grade 1, whereas 20% were classified as grade 2. Five grade 3 toxicities (fatigue, nail disorders, muscle spasms and diarrhoea) were reported by three patients in the 90-mg (30 mg t.d.s.) cohort. All of these events resolved, although two of the three patients required that study drug be permanently discontinued. No grade 4 events were reported and there were no deaths on study.

Two events considered to be DLTs (grade 3 fatigue, and concomitant grade 3 diarrhoea and intermittent muscle cramps) were reported by two patients in the 90-mg (30 mg t.d.s.) cohort. Thus, 60 mg (20 mg t.d.s.) was determined to be the MTD. The DRTs were seen in the 60-mg (20 mg t.d.s.) and 90-mg (30 mg t.d.s.) cohorts. Fatigue was the most commonly reported DRT.

A total of five serious adverse events were reported by five patients, only one of which was considered to have a potential causal relationship to sodium selenate. This was a patient in the 90-mg (30 mg t.d.s.) cohort who presented with an elevated creatinine level of 260 mmol l^–1^ (as compared with 90 mmol l^–1^ at screening) when scheduled biochemistry assessments were performed at the end of the first cycle of treatment. At this visit, the patient was also noted to have decreased bicarbonate levels (19 mmol l^–1^ compared with 30 mmol l^–1^ at screening), associated with diarrhoea and dehydration. The study drug was stopped immediately and the patient was admitted for investigation of acute renal impairment. Examination of the patient's clinical records revealed the patient had a history of proteinuria, macro-haematuria and underlying kidney disease before commencing sodium selenate. Despite investigation, no cause for the acute deterioration in renal function could be determined, and a contribution of sodium selenate to the existing underlying renal disease cannot be ruled out. Creatinine levels were further elevated to 290 mmol l^–1^ when re-tested 12 weeks after the last study dose, although bicarbonate levels had normalised. The four serious adverse events considered not to be associated with the study drug were episodes of pelvic pain, bowel obstruction, renal colic and lower respiratory tract infection.

Apart from the patient described above, there were no clinically significant laboratory results considered to be potentially related to the study drug, and no patients were found to have vital sign assessments or ECG results that were abnormal and clinically significant.

### Pharmacokinetic and metabolic profile

The PK parameters are detailed in Table 4. Sodium selenate exhibited linear pharmacokinetics, in terms of AUC *vs* dose, across the dose ranges assessed in this study, with the caveat of small cohort size. The selenate *t*_1/2_ ranged from 1.2 to 2.9 h in the 5–30 mg single-dose cohorts, respectively. The short half-life necessitated a three times a day daily dosing regimen to maintain adequate blood levels of sodium selenate. Peak selenate plasma concentrations were achieved within 1–4 h across all dose levels. At the recommended phase II dose, that is, 20 mg t.d.s., the mean *t*_max_ was 2.5 h and the mean *t*_1/2_ was 2.9 h.

The major selenium metabolite identified in plasma was selenite. Its plasma concentrations showed signs of accumulation, with *t*_max_ ranging from a mean of 90 h for the 10 mg t.d.s. cohort to 294 h for the 30 mg t.d.s. cohort (data not shown). Plasma selenite levels reached steady-state levels by 3 weeks. The ratio of selenite AUC_504_ *vs* selenate AUC_504_ ranged from 1.3 × at 10 mg t.d.s. to 7 × at the 30 mg t.d.s. dose level. Seleno-methionine, seleno-cyanate and methyl selenium species were other significantly minor metabolites identified in plasma. For the t.d.s. dosing cohorts, 16–37% of total daily dose of selenium was recovered in the 24-h urine. Selenate was the major selenium species identified in the 24-h urine accounting for 10–24% (based on the mean for each t.d.s. dosing cohort) of total daily selenium administered. Seleno-methionine and miscellaneous methyl selenium species were also major selenium compounds identified in 24-h urine; however, levels varied significantly across the patients in the t.d.s. dosing cohorts. Selenite was barely detectable in the urine, being <0.3% of total daily dose.

### Efficacy

**Figure 1:**
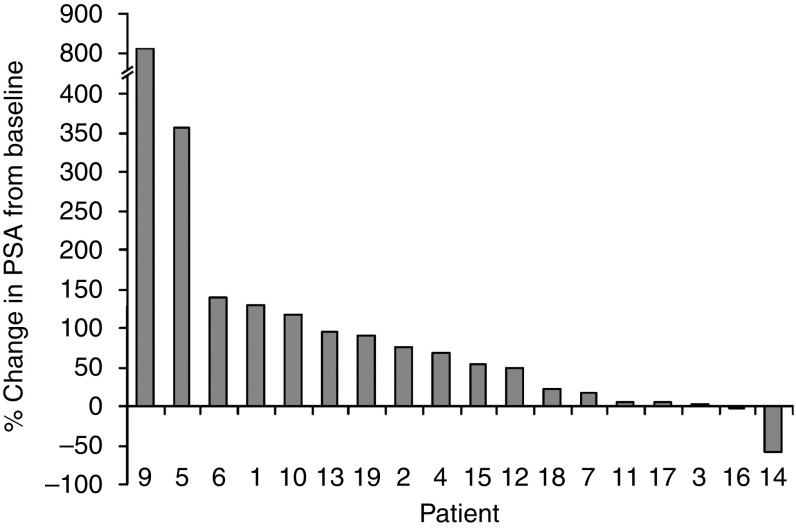
Waterfall plot of percentage change in prostate-specific antigen (PSA) from baseline to the end of the 12-week evaluation period (or withdrawal if earlier) for each patient.

Although the study was not designed to determine clinical efficacy, PSA was monitored throughout the trial as a surrogate marker of tumour response. The percentage change in PSA from baseline for each individual patient is shown in Figure 1. One patient from the 60-mg (20 mg t.d.s., patient 14) cohort experienced a significant PSA decline (maximal change in PSA from baseline −57.4%) that was maintained for 11 weeks and two patients (both from the 30-mg (10 mg t.d.s.) cohort) had disease stabilisation lasting 28 and 41 weeks, respectively. For those patients who completed the initial 12-week evaluation (*n*=12), median time to progression was 14.2 weeks (13.1–41.1)

**Figure 2:**
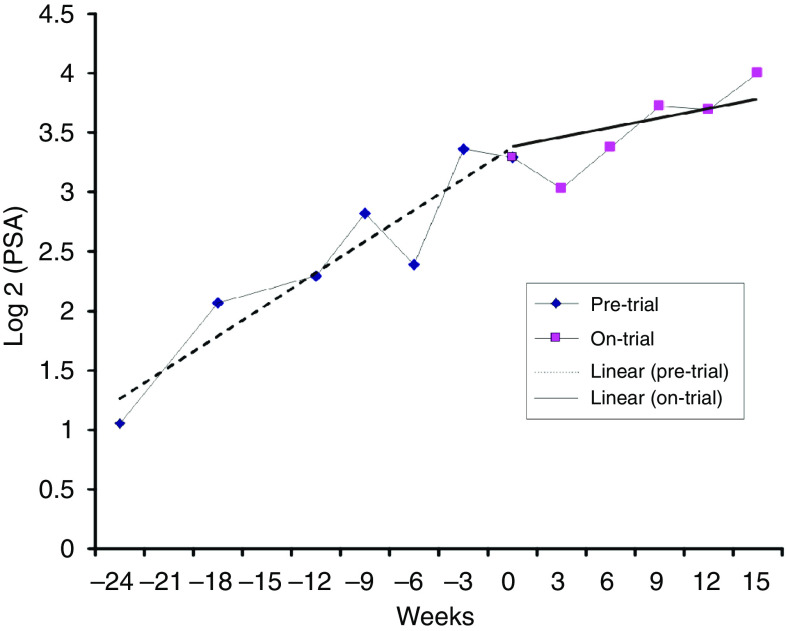
Prostate-specific antigen (PSA) doubling time before trial and during the main treatment phase.

Mean doubling time increased from 2.2 months (1.1–9.0) before trial to 3.9 months (1.1 to −2.0) during the main treatment phase (Figure 2).

## Discussion

The management of metastatic prostate cancer has changed significantly in the past decade. Cytotoxic chemotherapy with docetaxel is a standard of care in castrate-resistant disease and new therapies, including hormone agents such as abiraterone, show promising activity. However, no current therapy is curative and novel therapies are still needed. Most recently, many clinical trials have focussed on anti-angiogenic agents targeting critical kinases.

We have previously identified that the specific selenium-containing compound, sodium selenate, significantly boosts the activity of the protein phosphatase PP2A, inhibits VEGF-induced growth and survival signalling in endothelial cells, and impedes tumour neovascularisation.

This ‘first-in-human’ study examined the use of sodium selenate in men with CRPC before they received cytotoxic chemotherapy. Sodium selenate was well tolerated at doses up to 45 mg per day t.d.s. and the MTD was attained at a dose of 60 mg per day t.d.s. DLT was fatigue and diarrhoea, while other frequently reported adverse events included, nail disorders, muscle spasms, alopecia and nausea. Some of the side effects observed at the higher doses, in particular the fatigue, diarrhoea, alopecia and vomiting are perhaps attributable to the accumulation of the inorganic metabolite selenite, a selenium compound with marked cytotoxic properties and a very different pharmacokinetic profile compared with selenate, as confirmed in this study (Lu *et al*, 2009).

The study was not designed to evaluate the efficacy of sodium selenate. However, one PSA response was observed and two other patients appeared to have some stabilisation of disease. This modest activity is consistent with single-agent studies of other anti-angiogenic agents, including bevacizumab, sorafenib and sunitinib (Reese *et al*, 2001; Chi *et al*, 2008; Dror Michaelson *et al*, 2009).

On the bases of these observations, it would be reasonable to question the importance of angiogenesis to the development of prostate cancer. However, the continual development of a new vascular network is required to support the growth and increase in size of all solid tumours. The density of the vascular network that is established in any given tumour might reasonably be expected to reflect the growth, progression and metastatic potential of that tumour (Folkman, 1971), and indeed, tumour vascularity as measured by MVD has been shown to be a prognostic factor in a range of solid malignancies including that of prostate cancer (Weidner *et al*, 1991; Weidner, 1993; Weidner and Folkman, 1996; Strohmeyer *et al*, 2000).

Most of the evidence linking angiogenesis with prostate cancer progression has come from studies evaluating total MVD in prostate tumour samples, which almost invariably demonstrate a positive correlation with other histological features of tumour aggression and/or adverse clinical outcomes. However, as previously proposed, MVD may be poor surrogate of a tumour angiogenic potential, rather more accurately reflects tissue metabolic activity (Hlatky *et al*, 2002). Recent findings in renal cell carcinoma suggest that the relative degree of vessel differentiation within tumours correlates more closely with angiogenic activity than total MVD, with high densities of undifferentiated vessels significantly associated with higher tumour grades and shorter patient survival, in contrast to high densities of differentiated, vessel which correlated significantly with lower tumour grade and longer survival (Yao *et al*, 2007). Similar findings have been reported in prostate cancer samples, with significantly higher levels of proliferating immature tumour vessels identified in castration-resistant or metastatic tissue compared to organ confined cancers or benign tissue (Gravdal *et al*, 2009). More importantly, higher levels of immature vessels in radical prostatectomy specimens were a strong and independent predictor of biochemical failure, clinical recurrence or the development of bony metastases in multivariate analyses, confirming the clinical importance of angiogenesis in prostate cancer progression.

The expectation that single-agent anti-angiogenic therapy will result in the same type of clinical responses that are observed with traditional cytotoxic agents is based on the assumption that either the tumour itself, or the vascular network that supports it, is critically dependent on the signalling pathway inhibited. However, with the notable exception of clear cell renal carcinoma (CCRCC), significant or sustained responses are rarely observed with single-agent therapy. This clinical difference may simply reflect the strength of angiogenic drive within different tumour types, as renal cancers, together with glioblastomas, have the highest rates of endothelial cell proliferation and levels of immature blood vessels of tumour types studied, certainly significantly higher than prostate cancer, and thus may be more intrinsically sensitive to angiogenic inhibition (Eberhard *et al*, 2000). In addition, given the high frequency of VHL mutations in CCRCC, angiogenesis may be much more dependent on VEGF signalling than in prostate cancer, in which multiple redundant pathways have been implicated (Gravdal *et al*, 2006; Kim *et al*, 2006; Bergers and Hanahan, 2008; Yang *et al*, 2008). In future studies, the identification of prostate cancer patients most likely to respond to anti-angiogenic therapies, either by measuring levels of immature vessels in pretreatment biopsies, or by global markers of vessel turnover such as with circulating endothelial cells or their progenitors, may be important (Georgiou *et al*, 2008).

Despite their lack of single-agent activity, anti-angiogenic agents have shown favourable effects in boosting the efficacy of standard chemotherapy regimens in different tumour types (Ellis and Hicklin, 2008). Interestingly, this effect may be independent of a direct effect on new blood vessel formation, but more reliant on a relative normalisation of the vascular network and blood flow within tumours, improving delivery of chemotherapy. Disappointingly however, the recently completed but yet to be reported CALBG phase III study of docetaxel±bevacizumab (CALGB 90401) in men with CRPC failed to demonstrate an improvement in survival compared with chemotherapy alone (http://www.roche.com/media/media_releases/med-cor-2010-03-12.htm). As only the VEGF signalling pathway was targeted, it is possible that combining anti-angiogenic agents with different mechanisms of action can overcome the inherent redundancy in angiogenic signalling in prostate cancer, and improve clinical response rates. This is supported by recently published phase II data exploring the combination of docetaxel, bevacizumab, thalidomide and prednisone, with 90% of patients experiencing a PSA decline of ⩾50% (Ning *et al*, 2010).

Further exploration of sodium selenate as a single-agent in CRPC is not warranted; however, its combination with conventional cytotoxics in particular docetaxel, as well as with other anti-angiogenics with different modes of action is worthy of further exploration.

## Figures and Tables

**Table 1:** Patient characteristics

*n*	19
Mean age (range), years	72 (58–78)
Caucasian, *n* (%)	19 (100)
Mean PSA (range), *μ*g l^–1^	22 (7.5–245.1)
Mean PSAdt (range), months	2.18 (1.05–8.94)
Bone metastases (%)	10 (52.6)
Mean haemoglobin (range), g l^–1^	137.6 (109–166)
Mean alkaline phosphatase (range), U l^–1^	126.6 (59–491)
	
*WHO performance status*	
0	15
1	4
Previous therapy (%)	
Radical prostatectomy	3 (15.8)
EBRT	4 (21.1)
Median Gleason score (range)	8 (6-10)

Abbreviations: EBRT=External beam radiotherapy; PSA=prostate-specific antigen; PSAdt=PSA doubling time; WHO=World Health Organisation.

**Table 2:** Dose escalation and treatment completion

**Dose**	***N***	**Completed 12-week treatment**	**Reason for withdrawal**
5 mg per day	1	Yes	
10 mg per day	1	Yes	
15 mg per day	1	Yes	
30 mg per day	1	Yes	
30 mg (10 mg t.d.s.)	3	No	Disease progression
		Yes	
		Yes	
45 mg (15 mg t.d.s.)	2	Yes	
		Yes	
60 mg (20 mg t.d.s.)	6	No	Disease progression
		No	Disease progression
		No	Disease progression
		Yes	
		Yes	
		No	Adverse event (renal impairment)
90 mg (30 mg t.d.s.)	4	No	DLT (grade 3 fatigue)
		Yes	
		Yes	
		No	DLT (grade 3 diarrhoea and muscle spasms)

Abbreviation: DLT=dose-limiting toxicity.

**Table 3:** Toxicity experienced by ⩾2 patients thought to be at least possibly related to the study drug

**SOC**	**Preferred term**	**Grade 1/2 (%)**	**Grade 3 (%)**
Gastrointestinal disorders	Constipation	4 (21)	0 (0)
	Diarrhoea	4 (21)	1 (5)
	Nausea	5 (26)	0 (0)
	Retching	2 (10)	0 (0)
	Vomiting	4 (21)	0 (0)
General disorders	Fatigue	8 (42)	1 (5)
Metabolism and nutrition	Decreased appetite	5 (26)	0 (0)
Musculoskeletal and connective tissue disorders	Arthralgia	2 (10)	0 (0)
	Muscle spasms	8 (42)	1 (5)
	Myalgia	3 (16)	0 (0)
Nervous system	Dizziness	4 (21)	0 (0)
	Headache	3 (16)	0 (0)
	Hypoaesthesia	2 (10)	0 (0)
	Lethargy	5 (26)	0 (0)
Skin	Alopecia	8 (42)	0 (0)
	Nail disorders	5 (26)	1 (5)
	Pain of skin	2 (10)	0 (0)

**Table 4:** Pharmacokinetic parameters for selenate for all patient cohorts

	**AUC_0–24 h_** **(ng** **h** **ml^–1^)**	**AUC_0–168 h_** **(ng** **h** **ml^–1^)**	**AUC_0–336 h_** **(ng** **h** **ml^–1^)**	**AUC_last_** **(ng** **h** **ml^–1^)**	***T*_1/2_** **(h)**	***T*_max (0−last)_** **(h)**	***C*_max (0−last)_** **(ng** **ml^–1^)**	***C*_min (0−last)_** **(ng** **ml^–1^)**
*Single daily dose (mg)*								
5	—	—	—	126.9	1.23	1	56	2.78
10	—	—	—	8311.9	1.61	1	112	30.6
15	—	—	—	440.8	1.38	0.7	154	40.3
30	—	—	—	666.4	2.9	2.2	166	65.3
								
*Mullti-dose*								
*30 mg (10 mg t.d.s.)*								
*n*	3	3	3	3	3	3	3	3
Mean	502.39	1290.5	2049.3	2763.37	1.72	1.43	98.13	2.86
s.d.	161.92	293	426.7	972.46	0.25	0.6	56.36	0.91
CV %	32.23	22.7	20.8	35.19	14.8	42.05	57.43	31.85
								
*45 mg (15 mg t.d.s.)*								
*n*	2	2	2	2	2	2	2	2
Mean	549.1	2232.2	8437.1	15130.3	2.14	169.6	107.5	6.63
s.d.	17.5	92.8	6884.9	13931.5	1.34	237.1	19.2	1.99
CV %	3.2	4.2	81.6	92.1	62.6	139.8	17.8	29.99
								
*60 mg (20 mg t.d.s.)*								
*n*	6	6	6	6	6	6	6	6
Mean	1232.9	7201.1	11678.7	14354.7	3.09	2.6	165	11.23
s.d.	684	5892.2	9275.6	9632	1.04	1.2	72.3	5.57
CV %	55.5	81.8	79.4	67.1	32.5	46.1	43.9	49.61
								
*90 mg (30 mg t.d.s.)*								
*n*	4	4	3	3	4	4	4	4
Mean	1313.6	9783.1	15921.1	21521.4	2.26	13.4	218.8	18.63
s.d.	163.7	1862.6	3671.2	6843.7	0.58	23.5	90.7	8.73
CV %	12.5	19	23.1	31.8	25.7	174.7	41.5	46.87

Abbreviation: CV=coefficient of variance.
